# Music Stimulation for People with Disorders of Consciousness: A Scoping Review

**DOI:** 10.3390/brainsci11070858

**Published:** 2021-06-28

**Authors:** Giulio E. Lancioni, Nirbhay N. Singh, Mark F. O’Reilly, Jeff Sigafoos, Lorenzo Desideri

**Affiliations:** 1 Department of Neuroscience and Sense Organs, University of Bari, 70121 Bari, Italy; 2 Department of Psychiatry, Augusta University, Augusta, GA 30912, USA; nirbz52@gmail.com; 3 College of Education, University of Texas at Austin, Austin, GA 78712, USA; markoreilly@austin.utexas.edu; 4 School of Education, Victoria University of Wellington, Wellington 6012, New Zealand; jeff.sigafoos@vuw.ac.nz; 5 Department of Psychology, University of Bologna, 40127 Bologna, Italy; lorenzo.desideri2@unibo.it

**Keywords:** disorders of consciousness, unresponsive wakefulness syndrome, vegetative state, minimally conscious state, music, rehabilitation

## Abstract

Music stimulation is considered to be a valuable form of intervention for people with severe brain injuries and prolonged disorders of consciousness (i.e., unresponsive wakefulness/vegetative state or minimally conscious state). This review was intended to provide an overall picture of work conducted during the last decade to assess the impact of music on behavioral and non-behavioral responses of people with disorders of consciousness. Following the PRISMA-ScR checklist, a scoping review was carried out to identify and provide a synthesis of eligible studies published in English during the 2010–2021 period. Three databases (i.e., PubMed, PsycINFO, and Web of Science) were employed for the literature search. Thirty-four studies met the inclusion criteria. Those studies were grouped into three categories based on whether they assessed the effects of: (i) recorded music, (ii) interactive music, or (iii) response-contingent music. A narrative synthesis of the studies of each of the three categories was eventually provided. While the studies of all three categories reported fairly positive/encouraging results, several methodological questions make it difficult to draw conclusions about those results and their implications for intervention programs in daily contexts.

## 1. Introduction

Disorders of consciousness, such as unresponsive wakefulness syndrome (UWS) or vegetative state (VS) and minimally conscious state (MCS), can be long-lasting conditions (i.e., can remain largely unchanged for months and years), degrading the status of the persons affected and posing a great burden on their families and caregivers [1,2,3]. Given the damaging implications of those conditions, a variety of behavioral and non-behavioral interventions aimed at improving those conditions (i.e., increasing the level of consciousness, enhancing the quality of life, and creating new recovery perspectives) have been evaluated over the years [4,5,6].

Among the non-behavioral interventions, one can include the use of pharmacological agents (e.g., Amantadine [7,8]) and of forms of transcranial stimulation such as transcranial direct current stimulation [9,10] and repetitive transcranial magnetic stimulation [11,12]. Among the behavioral interventions, one can include several forms of music presentation, multisensory stimulation procedures, and messages with an affective-emotional content read to the patient by a family member [6,13,14,15].

Out of the aforementioned behavioral interventions, music stimulation is certainly the most popular and probably most widely credited approach [16,17,18,19,20]. The use of music has a long tradition with healthy and clinical populations and is considered to be associated with a range of psychological and physical benefits such as reduction of pain, anxiety, and restlessness/agitation in those populations [21].

Two of the reasons most frequently provided to recommend music as an intervention strategy to be adopted for people with disorders of consciousness may be of note. The first reason is that music is thought to affect neural networks, accelerate brain plasticity, and avoid sensory deprivation [22,23]. More specifically, music stimulation of the auditory area is expected to increase the activity in frontal, temporal, parietal, and subcortical regions with presumably important positive implications for the participants’ recovery process [18,24,25]. The second reason is the view that the emotional content of salient music can activate limbic and paralimbic structures, as well as the reward circuit, thus improving the general functioning and well-being of the people exposed to it [26,27]. Another possible motive that seems to justify the preference for music (or auditory stimulation in general over visual stimulation) is that people with disorders of consciousness tend to have an undamaged hearing system, as opposed to a frequently impaired visual system [17,19,28].

Given the importance attributed to music in interventions with people with disorders of consciousness, a variety of studies have been conducted to investigate its usability and impact [22,26,29,30,31,32]. Efforts have also been made to summarize the evidence available in the area. For example, Grimm and Kreutz [21] carried out a review of articles examining behavioral and neurological responses associated to music presentation. While they covered literature in English, German, and French from 1983 to April 2017, their article selection was restricted to studies on adults and excluded studies with less than three participants. More recently, Li et al. [18] carried out a review of studies published from 2004 to July 2018, but apparently adopted restrictive inclusion criteria. As a result, only 10 studies published between 2010 and 2018 were included in their review and one of the studies concerned the use of music with persons affected by mild traumatic brain damage. Finally, Grimm and Kreutz [33] provided a review of eight qualitative studies carried out from 1990 to 2015, contributing to extend the narrative surrounding the music approach more than the scientific evidence about it.

The present paper was to provide a review of the studies carried out between January 2010 and April 2021 and complement and integrate the aforementioned review articles published in the area. In fact, the new review (a) included studies published between 2017–2018 and 2021 (i.e., articles published during the time interval, which had not been covered by the previous reviews), and (b) reported single-case studies as well as studies using music contingent on patient’s responding (i.e., music that switched on as a consequence of responding) [34,35,36]. The latter studies (with the possible exception of [37]) had been omitted by all previous reviews. In essence, the new review was intended to provide a comprehensive picture of work conducted during the last decade to assess the impact of music on behavioral or non-behavioral responses of people with disorders of consciousness. Providing such a general picture to professionals working in the area was considered useful to update their knowledge and stimulate their research initiatives to add new evidence that could lead to better results in the efforts to help people with disorders of consciousness.

## 2. Materials and Methods

### 2.1. Search Strategy

We conducted a literature search following the guidelines provided by Preferred Reporting Items for Systematic Reviews and Meta-Analyses Extension for Scoping Reviews (PRISMA-ScR) [38]. A scoping review approach was chosen, given that our aim was to examine how extensively the impact of music on people with disorders of consciousness has been investigated and reported [39,40].

The search was performed using three databases; that is, PubMed, Web of Science, and PsycInfo. The same key words were used for each database: ‘vegetative state’ OR ‘minimally conscious state’ OR ‘post-coma’ OR ‘disorder of consciousness’ OR ‘unresponsive wakefulness’ OR ‘brain injury’ AND ‘music’ OR ‘environmental stimulation’.

**Figure 1:**
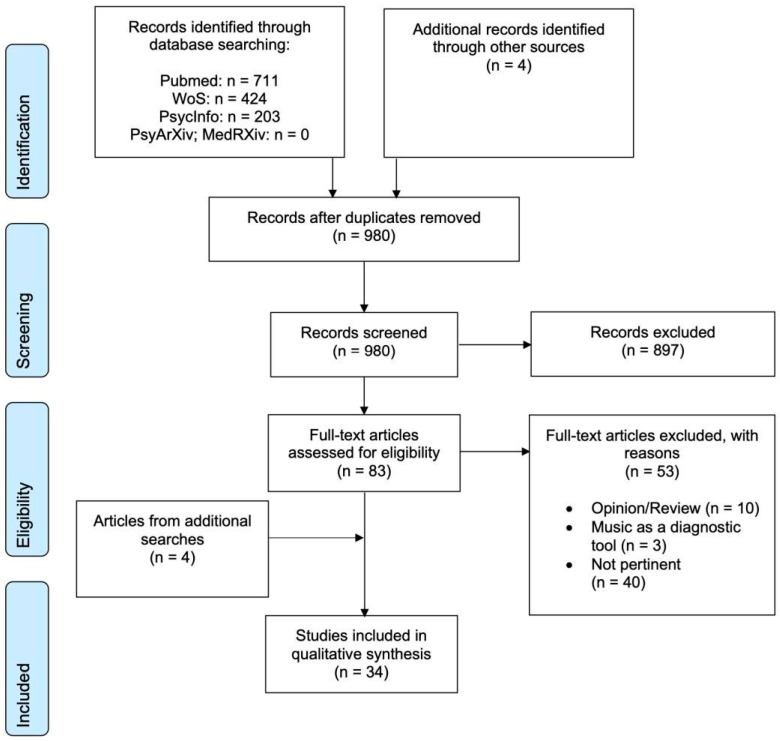
Flowchart of the literature search process.

The aforementioned databases and key words were chosen following consensus among authors. The search resulted in a total of 1338 papers. The number of papers was reduced to 980 after duplicates were removed. Figure 1 illustrates the search process and outcome. Initially, titles and abstracts of the 980 papers were screened. When the titles and abstracts were judged to be in line with the inclusion criteria (see below), the corresponding full-text articles were downloaded. Following this process, 83 full-text articles were downloaded. Those full-text articles were then read by the first and last author and 30 of them were found eligible for the review.

Four additional search strategies were also employed as supplements to the original/main search. First, the references of the 30 articles selected, as well as the references of recent review and opinion articles, were inspected. Second, a Google Scholar ‘cited by’ search was conducted using the aforementioned 30 articles. Third, a search alert in Google Scholar using the terms ‘minimally conscious state’, ‘disorder of consciousness’, ‘vegetative state’ and ‘unresponsive wakefulness’ was activated so as to receive notification of new publications appearing during the time when this paper was being written. Fourth, a search was also performed in PsyArXiv and MedRXiv repositories to identify articles not yet indexed in any of the other databases inspected. The aforementioned strategies led to the finding of four extra articles, with the consequence that 34 articles were finally included in the review (see Figure 1). The inclusion of new articles was completed in April 2021.

### 2.2. Inclusion and Exclusion Criteria

The selection was restricted to articles written in English and published in peer-reviewed journals between January 2010 and April 2021. Selection was based on the following inclusion criteria. First, the articles/studies involved the participation of people who (a) had a diagnosis of vegetative state/unresponsive wakefulness syndrome (VS/UWS) or minimally conscious state (MCS) or (b) were reported to have Glasgow Coma Scale scores in the 3–8 range [41]. Second, the studies were focused on presenting music stimulation and determining its impact on behavioral or non-behavioral outcome measures. Third, the studies reported quantitative or mixed quantitative and qualitative data. Studies were excluded if they did not meet one or more of the aforementioned criteria (see e.g., [22,28,30,42,43,44,45,46]).

### 2.3. Data Extraction and Coding

A data charting form was jointly developed by the authors, who worked iteratively until agreement was achieved as to the most adequate set of information that should be reported for the single studies. Eventually, the data extracted for each study entailed (a) the year in which the study was published and the country in which it was carried out, (b) the patients involved, (c) the intervention/stimulation conditions available, (d) the assessment strategy applied (i.e., the protocol followed to assess the impact of music), (e) the measures recorded, and (f) the outcome. The patients’ disorders of consciousness and the outcome of the studies were, as far as possible, described using the same terminology as employed by the studies’ authors. In the end, following a consensus-based approach among authors, codes were created to group the studies included in the review according to the different music stimulation conditions they represented (see Results).

### 2.4. Interrater Agreement

Interrater agreement was checked between the first and last author (a) on scoring the eligibility of the 83 full text articles, which were downloaded after screening titles and abstracts, and (b) on reporting the data extracted from the articles reviewed (see Results and Tables 1–3). Interrater agreement on this latter measure was checked over 10 articles. The percentage of agreement on the 83 full text articles was 96%. That is, the authors provided the same label (i.e., “included” or “excluded”) for 80 of the 83 articles. Consensus between authors on the three articles with initial disagreement was then achieved after a brief discussion. Interrater agreement on reporting the data extracted from 10 of the articles included in the review was 100%.

## 3. Results

As indicated above, 34 studies were identified that assessed the impact of music on the condition of people with disorders of consciousness (see Table 1, Table 2 and Table 3 for a general view). Those studies were conducted in Italy (*n* = 10), China (*n* = 4), Germany (*n* = 4), France (*n* = 3), Belgium (*n* = 2), Brazil (*n* = 2), Argentina (*n* = 1), Australia (*n* = 1), Austria (*n* = 1), Iran (*n* = 1), Israel (*n* = 1), Japan (*n* = 1), South Korea (*n* = 1), Spain (*n* = 1), and the United Kingdom (UK) (*n* = 1). In total, 411 patients were involved. This number also includes patients exposed to control conditions. The studies were grouped into three categories: (i) studies assessing the effects of recorded music (i.e., music recorded via some electronic device and presented to the patient through such device), (ii) studies assessing the effects of interactive music (i.e., studies in which a music therapist was directly involved in producing/presenting the music and modulating it according to the patient’s situation), and (iii) studies assessing the effects of response-contingent music (i.e., music following the occurrence of specific behavioral or non-behavioral responses). Within each of the three categories, studies differed in terms of (a) the types of music presented to the patients and of the length of time the music was presented, (b) the types of measures recorded to determine the impact of music, (c) the characteristics of the people involved (i.e., the severity of their disorders of consciousness), and (d) the assessment strategies implemented (e.g., whether the impact of music was compared with a baseline/non-music condition or whether different forms of music or music and noise conditions were compared). Table 1, Table 2 and Table 3 present brief summaries of the studies carried out within the three categories mentioned above, respectively, and the text provides more specific descriptions of some of those studies. Such descriptions are intended to help the reader (a) determine the level of knowledge available as to the potential of music in intervention programs directed at people with disorders of consciousness, and (b) formulate suggestions for new research initiatives that could help fill some of the gaps in the existing knowledge.

### 3.1. Studies Assessing the Impact of Recorded Music

Nineteen of the 34 studies (including 343 patients) were carried out to assess the impact of recorded music. As shown in Table 1, recorded music could involve, among others, a preferred song or segment of it [41,42], a compilation of preferred music pieces [29], classic compositions [22,47], and folk pieces [32]. The music stimulation period could range from 30 s [48] to 1 h [49]. Moreover, the assessment procedure used in the studies could vary in terms of the intervention/stimulation (music and non-music) conditions included and the measures recorded to determine the impact of those conditions.

For example, Choudhry et al. [50] exposed three MCS patients to an assessment procedure that lasted 70 min. The procedure involved the presentation of three 10-min stimulation conditions consisting of preferred music, pink noise, and preferred music played backwards, respectively. The order of the stimulation conditions was randomized. Each condition was preceded and followed by a 10-min washout period. Throughout the 70-min assessment, recording occurred of the patients’ respiration frequency, heart rate, blood pressure, and behavioral responses (e.g., movements and eye openings). The findings showed that the patients tended to have an increase in blood and respiratory measures during the preferred music condition, but the increase did not reach statistical significance (i.e., contrary to the evidence obtained from healthy control participants). One patient also had an increase in behavioral responses.

Luauté et al. [26] carried out a study with 11 (5 UWS and 6 MCS) patients to compare the effects of preferred music with the effects of neutral sound, preferred odor, and neutral odor being presented to those patients. Within each of the four sessions implemented, all four stimulation conditions were available for 5-min periods. Two types of measures were collected; that is, the performance on items of the Coma Recovery Scale-Revised [51] following the different types of stimulation, and the electrodermal responses during the various types of stimulation. Yet only the data concerning electrodermal responses were reported. These data showed no significant differences between stimulation conditions. These findings contrasted with those obtained for healthy control participants whose responses during preferred music were significantly different from their responses in the other conditions.

Wu et al. [32] exposed 14 patients (seven with a diagnosis of UWS and seven with a diagnosis of MCS) to three different stimulation conditions; that is, folk music, a family member calling the patient’s name, and white noise. Each stimulus condition lasted 5 min. The conditions’ order of presentation was randomized across patients with a washout period of 2 min between conditions. The patients’ brain response to the three stimulation conditions and a baseline (silence) state was monitored by quantitative electroencephalography. Data showed that the condition in which the patient was called by name produced the largest impact, while white noise and music activated more areas of the cerebral cortex than the baseline silence state. The differences between music stimulation and silence regarding delta and theta/beta values showed marginal statistical significance for MCS patients but not for UWS patients.

Boltzmann et al. [28] exposed 13 UWS patients to two stimulation conditions, one consisting of a preferred music compilation and the other consisting of an aversive auditory stimulation. The two stimulation conditions and scanner noise, each lasting 8 min, were presented in a random order across patients. During aversive auditory stimulation, functional connectivity was increased to the right insular cortex and the cingulate gyrus in the auditory network over the levels observed during scanner noise. During preferred music, functional connectivity to the right planum polare was increased over the levels observed with scanner noise. In essence, preferred music and aversive auditory stimulation modulated activity of the auditory network, suggesting that these types of stimuli may be useful for helping UWS people.

Zhang et al. [52] carried out a study involving 20 MCS patients who were divided into two groups of 10. Each patient received auditory stimulation for 30 min, five times per week for 6 weeks. The stimulation for one of the two groups was chosen by a music therapist and included pieces of Chinese or Japanese composers and was characterized by clear and simple melody. The stimulation for the other group was chosen by the patients’ families and mostly consisted of popular songs or folk music. The results reported for the two groups (a) suggested that the therapist-selected music stimulation produced a significantly higher interactive activity of the autonomic nervous system and (b) implied that such types of stimulation may be viewed as more suitable to arouse the level of responsiveness of patients with disorders of consciousness.

**Table 1:** Studies Assessing the Impact of Recorded Music.

Studies and Countries of Origin	Patients	Stimulation Conditions	Assessment Protocol	Measures Recorded	Outcome
Riganello et al. (2010), Italy [47]	Nine VS patients. Age: 16–48 years	Four different pieces of classic music of near 4 to over 10 min	All pieces were presented, two pieces per session, with an interval of 10 min between pieces	Physiological indices	Data showed that different types of music caused changes in heart rate variability compatible with residual emotional reactions
Puggina et al. (2011), Brazil [53]	30 coma-VS patients. Mean age: 46.2 years	Preferred song and a 3-min family message	15 patients received the two types of stimulation on each of 3 days, the other 15 patients did not receive stimulation	Physiological indices and behavioral responses	Some significant changes in facial expressions with both types of stimulation and some changes in physiologic indices with the message
Okumura et al. (2014), Japan [48]	Seven VS or MCS patients. Age: 16–55 years	Music from ‘Les Toreador’, and two general sound conditions in segments of 30 s	The patients were exposed to sound conditions and ‘Les Toreador’	Brain activity (fMRI)	Music activated the bilateral superior temporal gyri for the two MCS patients and only one of the five VS patients.
Ribeiro et al. (2014), Spain [54]	26 VS patients. Age: 54.1 years	20-min pieces of classic relaxing music and relaxing music with nature sounds, and 1-h periods of radio music	13 patients were exposed to 18 music sessions, three sessions per condition, the other 13 patients did not receive any stimulation	Physiological indices and behavioral responses	Significant differences were observed between the patients who received stimulation and the others. Changes concerned physiological indices (radio music) or both measures (the two relaxing music conditions)
Castro et al. (2015), France [42]	13 VS or MCS. Mean age: 41.5 years	1-min segments of preferred music or of music-like noise	Segments of preferred music and of music- like noise preceded presentations of name sequences (including the patient’s own name and other seven names)	Brain activity (EEG)	Discriminative cerebral responses to the patient’s own name occurred more frequently after the presentation of preferred music segments
Heine et al. (2015), Belgium [29]	Five UWS or MCS patients. Mean age: 50 years	10-min compilation of preferred music and repetitive noise	Patients were exposed to the two types of stimulation in different order, with a 10-min interval separating them	Brain activity (fMRI)	Stronger functional connectivity with the left precentral gyrus and the left dorsolateral prefrontal cortex was shown during music
Puggina & da Silva (2015), Brazil [41]	39 coma-VS patients. Age: ≥ 18 years	A preferred song, a message read by a family member, or silence, each lasting 2–4 min	Patients were divided into three groups, each exposed to one of the aforementioned conditions over two sessions	Behavioral responses and physiological indices	Music produced more/significant changes in physiological indices while message produced more significant changes in behavioral (facial) responses
Riganello et al. (2015), Italy [22]	Nine UWS patients. Age: 16–48 years	Four different pieces of classic music	All pieces were presented, two pieces per session, with an interval of 10 min between pieces	Physiological indices	Variations in physiological responses were observed in relation to specific music parameters
Sun & Chen (2015), China [55]	40 coma-VS patients. Age: 18–55 years	30-min sessions of preferred music	20 patients received the music sessions four times a day for 4 weeks. The other 20 patients received no music	Brain activity (EEG) and behavioral responses	Final assessment showed that patients who received music had significantly higher Glasgow Coma Scale scores and significantly lower Quantitative EEG values
Choudhry et al. (2016), Germany [50]	Three MCS patients. Mean age: 58 years	10-min segments of preferred music, of pink noise, and of preferred music played backwards	Patients received the stimulation segments in different order, with washout periods separating the segments	Physiological indices and behavioral responses	No significant changes (but trends) were found on both the indices and responses
Park et al. (2016), South Korea [49]	14 VS patients. Age: 19–61 years	1-h periods of preferred music and of relaxation music	The patients received both stimulation periods according to a cross-over design with a washout day separating them	Behavioral responses	There was a significant decline in agitation during the preferred music period. No decline was observed after the end of this period or during the relaxation music period
Heine et al. (2017), France [30]	13 UWS or MCS patients. Age: 23–63 years	5-min stimulation sessions with preferred music, neutral sound, and preferred and neutral olfactory stimuli	After each stimulation session, patients were presented one of four items of the Coma Recovery Scale-Revised	Behavioral responses	Preferred music led to higher levels of performance on the scale items than any of the other stimulation conditions
Luauté et al. (2018), France [26]	11 UWS or MCS patients. Age: 23–63 years	5-min stimulation sessions with preferred music, neutral sound, and preferred and neutral olfactory stimuli	During each stimulation session, recording occurred of the patients’ skin conductance response	Physiological indices	No significant differences between stimulation conditions were detected
Li et al. (2018), China [56]	19 VS or MCS patients with previous alcohol or smoke addiction. Age: 45 years	90 s of Chinese classic music, 90 s with a family member calling the patient’s name, 36 s of wiping alcohol on the participant’s lips, and 36 s with a cigarette smell	EEG signals were recorded during rest and during stimulation. All types of stimulation were presented in different order and interspersed with no stimulation periods	Brain activity (EEG)	The highest level of EEG response was related to calls of the patient’s name followed by the alcohol and smoke stimulation and the music. The differences were statistically significant
Wu et al. (2018), China [32]	14 UWS or MCS patients. Age: 19–70 years	5-min sessions with folk music, family members calling the patient’s name, white noise, and baseline silence	Patients received all 3 types of stimulation according to a different order and separated by a 2-min washout period	Brain activity (EEG)	Cerebral activation was higher when the patient was called by name. The difference between music and silence showed marginal statistical significance for MCS patients but not for UWS patients
Carrière et al. (2020), Belgium [57]	Four UWS or MCS patients. Age: 24–50 years	Patient’s preferred music and a non-stimulation condition	Patients were exposed to both conditions in random order with a washout interval between them	Brain activity (fMRI)	Increases in connectivity were observed during music stimulation in brain regions involved in consciousness, language, emotion, and memory processing
Boltzmann et al. (2021), Germany [28]	13 UWS patients. Age: 44–77 years	8-min segment of preferred music and 8-min segment of aversive auditory stimulation	Patients received the two types of stimulation and scanner noise in counterbalanced order	Brain activity (fMRI)	Functional connectivity of the auditory network was modulated by preferred music and aversive auditory stimulation
Yekefallah et al. (2021), Iran [58]	54 UWS patients. Mean age: 37.7 years	15-min sessions of melodic music	27 patients received seven music sessions while 27 other patients did not receive any stimulation	Behavioral responses	Level of consciousness, as measured via the Glasgow Coma Scale, increased from pre- to post-stimulation in five of seven evaluations
Zhang et al. (2021), China [52]	20 MCS patients. Mean age: 46 years	30-min sessions with therapist-selected music or family-selected preferred music	10 patients received therapist-selected music, and the other 10 family-selected preferred music over 6 weeks	Physiological indices	Therapist-selected music elicited a significantly higher interactive activity of the autonomic nervous system

Abbreviations: VS = Vegetative State; UWS = Unresponsive Wakefulness Syndrome; MCS = Minimally Conscious State; EEG = Electroencephalography; fMRI = Functional Magnetic Resonance Imaging.

### 3.2. Studies Assessing the Impact of Interactive Music

Six of the 34 studies (including 44 patients) were carried out to assess the impact of interactive music. As shown in Table 2, the studies varied in the way the interactive music therapy was applied, in the presence or absence of control stimulation conditions, and in the type of evidence recorded to document the effects of the music intervention on the patients’ responding.

For example, O’Kelly et al. [59] carried out a study with 21 patients, 12 with a diagnosis of VS and nine with a diagnosis of MCS, who were exposed to a 5-min baseline silence followed by four stimulation conditions. Those conditions included live performance of preferred songs with the therapist also calling the patient’s name, improvised music entrained to respiration, recordings of disliked music, and white noise. The order of the conditions was randomized across patients and a 2-min washout period separated them. Data showed that the patients presented large differences in responding. Even so, significant post hoc EEG amplitude increases were recorded during live performance of preferred music for frontal midline theta waves in six VS and four MCS patients, and frontal alpha waves in three VS and four MCS patients. Behavioral data showed a significant increase in blink rates during the same live music condition for VS patients.

Lichtensztejn et al. [44] intervened with a patient who had a diagnosis of VS. The intervention consisted of live music during which the patient was assisted by a family member in making minimal movements. Improvisation by the therapist involved the presentation of non-familiar musical styles as well as sharp rhythmic pauses at the end of each melodic phrase. The patient received a 30-min music therapy session per day, five days a week, over a one-month period. Data on the patient’s behavioral responding involved observational reports shared by therapists and family members, video-recordings as well as the patient’s performance on the Wessex Head Injury Matrix (WHIM), which is a 62-item scale aimed at assessing/monitoring recovery of cognitive functions [60]. Observational reports indicated an increase in responses such as head raising and other head movements. The score on the WHIM also showed an increase during the music therapy period.

Binzer et al. [61] assessed the impact of a live music improvisation technique known as “individual dialogic music therapy” with seven patients, four with a diagnosis of UWS and three with a diagnosis of MCS. Every patient was exposed to three 20-min stimulation conditions. The first and third condition consisted of environmental stimulation that did not involve any specific interaction with staff or other human agents. The second condition involved the presence of the music therapist who provided actively improvised music stimulation, such as singing, humming, or playing instruments. Improvisation began with simple vocal or instrumental sounds and melodies. Tempo and rhythm were synchronized with the patient’s respiration intervals. Video-recordings of the patients during the stimulation conditions were analyzed through a tool known as the “Music Therapy in a Vegetative or Minimally Conscious State (MUVES)”, which is intended to measure auditory, visual, motor and oral functions, as well as communication and vigilance. During the live music period, patients achieved significantly higher MUVES total scores than during the other conditions. The environmental stimulation condition that followed the music therapist’s improvisation period was always associated with the lowest scores.

**Table 2:** Studies Assessing the Impact of Interactive Music.

Studies and Countries of Origin	Patients	Stimulation Conditions	Assessment Protocol	Measures Recorded	Outcome
O’Kelly et al. (2013), UK [59]	21 VS or MCS patients. Age: 22–76 years	Live performance of preferred song, improvised melody plus patient’s name, recordings of disliked music, and white noise	Patients received each of the 4 types of stimulation presented in random order and separated by a 2-min washout	Physiological indices, brain activity (EEG), and behavioral responses	Live performance of preferred song was linked with significant post hoc increases for frontal midline theta or frontal alpha for most patients, and increases in eye blinks for VS patients
Bower et al. (2014), Australia [62]	One VS patient. Age: 10 years	Sessions of 5–22 min, with the therapist singing preferred songs and adapting the singing to the patient’s behavior	Videos of the sessions and pre- and post-session periods were analyzed for responses to music and agitation	Behavioral responses	The patients seemed to have high levels of reaction (e.g., acceptance and rejections) to music; no conclusions could be drawn about agitation
Lichtensztejn et al. (2014), Argentina [44]	One VS patient. Age: 22 years	A plurality of interactive music sessions with improvisation and possible family members’ participation	Observation of the patient’s behavior during the music periods and the administration of the Wessex Head Injury Matrix	Behavioral responses	The patient was reported to show multiple attention and participating responses during the music and a clear increase in the Wessex Head Injury Matrix scores
Raglio et al. (2014), Italy [63]	10 VS or MCS patients. Age: Not reported	Two cycles of 15 30-min sessions with the therapist providing the musical input and adapting it to the patient	Data recording occurred before and after each session as well as during the period separating the two session cycles	Physiological indices and behavioral responses	Some significant changes were observed in physiological indices for VS patients and in behavioral measures particularly for MCS patients.
Steinhoff et al. (2015), Austria [31]	Four UWS patients. Age: Not reported	15 music sessions of about 27 min with the therapist using various instruments and singing while adapting to the patient	Two patients were provided with the music sessions and two did only receive standard care	Brain activity (PET)	Substantial activity increase in frontal areas, hippocampus and cerebellum was reported only for the patients exposed to music
Binzer et al. (2016), Germany [61]	Seven UWS or MCS patients. Age: 22–69	20-min sessions, two of which involved basic environmental stimulation and one involved the music therapist interacting with and adapting the music to the patients	Every participant received all 3 sessions	Physiological indices and behavioral responses	Patients had significantly better performance scores on an evaluation tool focusing on vigilance, and sensory and communication responses

Abbreviations: VS = Vegetative State; UWS = Unresponsive Wakefulness Syndrome; MCS = Minimally Conscious State; EEG = Electroencephalography; PET = Positron Emission Tomography.

### 3.3. Studies Assessing the Impact of Response-Contingent Music

Nine of the 34 studies (including 21 patients) were aimed at assessing the impact of response-contingent music (see Table 3). In these studies, preferred music was delivered following the performance of a specific behavioral response (e.g., prolonged eyelid closure or finger movement) or brain activity (e.g., reduction of the theta/beta ratio values).

For example, Lancioni et al. [64] assessed whether response-contingent music stimulation would be effective in increasing the frequency of simple behavioral responses (i.e., full and protracted eyelid closures or finger movements) in three MCS patients. The study was carried out according to an ABAB (reversal) design and involved 5-min sessions. During the A (baseline) phases, the patients wore an optic or touch sensor that would detect their behavioral responses, but the responses were not followed by stimulation. During the B (intervention) phases, responses were followed by 15-s segments of preferred music and songs. Data showed that during the intervention phases, the patients had large and statistically significant increases in response frequency.

Lancioni et al. [65] carried out a study with two patients who had a diagnosis of VS. For each patient, an extended ABAB design was used. That is, the patient was initially exposed to baseline (A) non-stimulation phases alternated with intervention (B) phases in which each response was followed by 10 s of preferred music. Then, control phases were used in which the stimulation was available continuously throughout the sessions. All sessions were 5-min long and the type of response recorded during the sessions via an optic sensor was protracted eyelid closure. Data showed that both patients had large (statistically significant) increases in response frequency during the B phases as compared to the A phases. They also had a significant decline in responding during the control phases (in which music occurred regardless of the patients’ responses) as compared to the B phases (in which responses were needed to switch on music). This discrimination between the B phases and the control phases, with relative response adjustments, was taken as a sign of the patients’ environmental awareness and of their transition to a MCS.

Keller and Garbacenkaite [34] carried out a study involving three UWS patients. The patients received a daily session of neuro-feedback for 3 weeks. Specifically, the patients were presented with their preferred music whenever their theta/beta ratio values dropped below a specific threshold. In addition to the recording of these values, weekly assessments with the Coma Recovery Scale-Revised (CRS-R) were carried out. Data showed that the theta/beta ratio of the first patient decreased over time. A similar result was obtained with the second patient whose theta/beta ratio and theta amplitude decreased. In contrast with the first two patients, the third patient showed highly fluctuating amplitudes. The CRS-R scores for the first patient increased during the neuro-feedback intervention period and slightly decreased afterwards. The CRS-R scores of the second patient also increased during the intervention period and continued to rise slightly after the end of it. There were no changes in CRS-R scores for the third patient during or after the intervention period.

**Table 3:** Studies Assessing the Impact of Response-contingent Music.

Studies and Countries of Origin	Patients	Stimulation Conditions	Assessment Protocol	Measures Recorded	Outcome
Lancioni et al. (2010), Italy [64]	One VS patient. Age: 41 years	10- to 15-s segments of Preferred music which could be occasionally interspersed with familiar voices	The music segments were presented contingent on lip-movement responses during the intervention (B) phases of an ABAB design	Behavioral (lip movement) responses	The frequency of lip- movement responses during the B phases (when music followed each response) showed a large (statistically significant) increase
Lancioni et al. (2010), Italy [66]	Two MCS patients. Age: 53 and 56 years	10- to 15-s segments of preferred music	The music segments were presented contingent on finger and upward eyelid movements for the two participants, respectively, according to a multiple probe across responses design	Behavioral (finger and eyelid) responses	The frequency of the responses followed by preferred music showed a large and statistically significant increase
Lancioni et al. (2011), Italy [65]	Two VS patients. Age: 54 and 62 years	10-s segments of preferred music or uninterrupted music stimulation	The music segments were presented contingent on prolonged eyelid closures during the intervention (B) phases of extended ABAB designs. Control phases with music stimulation presented throughout the sessions were also used	Behavioral (eyelid) responses	The frequency of eyelid responses increased largely/significantly during the B phases as compared to the A (non-stimulation phases) and was higher than the frequency observed during the control phases
Lancioni et al. (2011), Italy [67]	Three MCS patients. Age: 67–77 years	10- to 15-s segments of preferred music	The music segments were presented contingent on prolonged eyelid closures or finger movements during the B phases of an ABAB design	Behavioral (eyelid and finger) responses	The frequency of the eyelid and finger responses increased largely/significantly during the B phases
Lancioni et al. 2012), Italy [43]	Two MCS patients. Age: 59 and 60 years	15-s segments of preferred music	The music segments were presented contingent on prolonged or repeated eyelid closures according to a multiple baseline design across participants	Behavioral (eyelid) responses	The frequency of the eyelid responses increased largely/significantly during the intervention with music stimulation
Lancioni et al. (2012), Italy [68]	One MCS patient. Age: 53 years	8-s segments of preferred music	The music segments were presented contingent on finger movements during the B phases of an ABAB design	Behavioral (finger) responses	The frequency of the finger responses increased largely/significantly during the B phases
Lancioni et al. (2012), Italy [69]	Four MCS patients. Age: 37–78 years	10- to 15-s segments of preferred music, which could be occasionally interspersed with familiar voices for two of the participants	The music segments were presented contingent on prolonged eyelid closures, finger movements, or big toe movements according to an ABAB design	Behavioral (eyelid, finger, and big toe) responses	The frequency of the patients’ responses followed by music increased largely/significantly during the B phases
Keller & Garbacenkaite (2015), Germany [34]	Three UWS patients. Age: 48–72 years	Preferred music	Preferred music was presented contingent on the theta/beta ratio level dropping below a certain threshold	Brain activity (EEG) and behavioral responses	Two of the patients showed a decrease in their theta/beta ratio and theta amplitudes and also some behavioral improvement as measured by the Coma Recovery Scale-Revised
Karpin et al. (2020), Israel [70]	Three MCS patients. Age: 20–66 years	30-s segments of preferred music	The music segments were delivered on the patients’ performance of small (e.g., eye closure) responses	Brain activity (EEG) and behavioral responses	Reports indicated that the patients were successful in acquiring the responses and maintaining adequate brain engagement

Abbreviations: VS = Vegetative State; UWS = Unresponsive Wakefulness Syndrome; MCS = Minimally Conscious State; EEG = Electroencephalograph.

## 4. Discussion

The use of music stimulation is certainly the most popular and probably most widely credited behavioral approach to help people with disorders of consciousness improve their condition and enhance their recovery perspectives. This review provides a picture of the different ways music has been presented to UWS/VS and MCS patients (i.e., recorded music, interactive music, and response-contingent music) and of the procedures implemented to determine its impact on the condition of those patients. The review includes 34 studies, many of which were not reported in previous literature reviews (i.e., studies from 2017–2018 to 2021, single-case studies, and studies using music contingent on patient’s responding). In essence, the present review extends and integrates previous literature syntheses in the area [18,21,33], and highlights that a wider range of approaches to music presentation is available for the treatment of UWS/VS and MCS patients. Previous reviews, for example, had neglected studies using music presentation contingent on patients’ responding, while this approach represents an additional intervention option for these patients.

It may be important at this point to discuss (a) the practical aspects/characteristics of the different music approaches and the results reported, (b) the measures recorded to assess the impact of those approaches, (c) the methodological conditions applied for the assessment, and (d) possible future directions.

### 4.1. Practical Aspects of the Different Approaches and Results Reported

With regard to this point, one can argue that the approach based on recorded music may be considered the simplest and most convenient of the three. It can be applied independent of any specific technology, environmental arrangement, or specialized personnel in virtually any context and by virtually any caregiver. As to its impact, the data of the studies reviewed seem to be fairly encouraging. Exceptions might be represented by the studies of Choundhry et al. [50]; Luauté et al. [26], which were described above, and to some extent the study of Li et al. [56]. Regarding the last study, the authors reported that the impact of music was smaller than that produced by calling the patient by name and that produced by stimuli related to the patients’ habits (i.e., alcohol or smoking). However, the authors did not clarify whether such impact was of any possible (practical) relevance.

The second approach (i.e., interactive music) is more elaborate and complex than the first (i.e., recorded music) and requires the presence of specialized personnel to be implemented. The results reported by the six studies adopting this approach appear to be rather positive. The two basic questions one may raise here are (a) whether this approach can be viewed as more effective than the first and (b) whether any possible advantage of interactive music over recorded music is due to the music variations presented by the therapist during the interactions with the patient or to a combination of music with non-music stimulation (e.g., the therapist calling the patient’s name and providing visual inputs [31,44]). These questions seem to be practically relevant and yet they are unanswered as no attempts were made to compare the effects of these two approaches.

The third approach is focused on determining whether music (a) can represent a reinforcing event for the patient and (b) can motivate the patient to perform/consolidate a specific response to access that event independently. Such an approach typically requires the use of technology to detect the behavioral or brain response targeted for the intervention and regulate the delivery of a specific period of preferred music for such response. The data of the nine studies using this approach provided a generally affirmative answer to the aforementioned questions thus confirming that music can be a reinforcing event and can help the patient learn to link the occurrence of such event to the response that precedes its occurrence. Learning such a link makes the response a purposeful requesting act.

### 4.2. Measures Used to Determine the Impact of Music

The measures used to determine the impact of music involved physiological indices, brain activity, and behavioral responses. Physiological indices typically included measures such as heart rate or heart rate variability, respiration frequency, blood pressure, and electro-dermal (skin) conductance (e.g., [22,26,47,50,54]. Brain activity was assessed via means such as magnetic resonance imaging (e.g., [28,29,57]), electroencephalographic recording (e.g., [34,42,56,59,70]), and positron emission tomography [31]. Behavioral responses included facial expressions and blinks [41,53,54,59], eye, lip, finger and/or head movements [43,50,64,67], or forms of agitation movements [49].

In light of the different measures used by the studies, two main considerations seem to be in order. First, it is difficult to compare the results reported when those results are based on different types of measures. Specifically, the positive evidence provided by a change in some physiological indices (e.g., heart rate, blood pressure, or respiration frequency) may not be considered equivalent to the evidence provided by changes in magnetic resonance imaging in spite of the tendency to consider physiological indices (a) meaningful representations of the patients’ alertness and attention to the stimuli and thus (b) suggestive of the patients’ recovery perspectives [71]. Second, it may also be difficult to compare the results of studies that apparently monitored the same types of responses but used different approaches. For example, the occurrence of behavioral responses (e.g., blinks or finger movements) in a study presenting periods of recorded music could be interpreted as a sign of alertness, possibly attention to the music. The occurrence/increase of behavioral responses in a study in which music is contingent on those responses may be interpreted as a sign of learning (i.e., as the patient’s ability to see the link between the two) and thus use the first as a means to (purposefully) access the second [35,72].

### 4.3. Methodological Aspects of the Assessment Process

An analysis of the methodological aspects (designs) involved in assessing the impact of music may be summarized in the following points. First, a large number of the studies using recorded music typically relied on (a) the presentation of the music condition in random order with the presentation of other stimulation (music or non-music) conditions [22,42,47,48,50], or (b) the presentation of the music condition(s) for the experimental group and not for the control group [52,53,54,55]. Often, healthy (comparison) participants were also involved in the studies and exposed to the same conditions as the patients. Second, the studies using interactive music relied mainly on (a) the presentation of various stimulation conditions to be compared [59,61] or (b) multisession music stimulation with sequential observations and assessment checks [44,62,63]. Third, the studies using response-contingent stimulation relied on (a) single-subject research designs alternating baseline and music intervention within or across patients [43,64,66,73] or (b) case series with a simple baseline-intervention sequence [70].

In light of the above, a number of considerations can be made. First, designs involving the presentation of various conditions in random order across participants or the use of randomly selected experimental and control groups may be considered fairly adequate from a methodological standpoint. In spite of that, the results of the studies using those designs could hardly be taken as cumulative evidence. In fact, (a) the music and non-music stimulation conditions being compared varied largely across studies, (b) the duration (presentation time) of those conditions also varied, and (c) the evidence reported was frequently based on different types of measures. All these aspects call for extreme caution in drawing conclusions about the results available. Second, designs involving multiple music sessions and sequential checks (or a final test) on the patients’ progress may not help the reader answer a basic/critical question, that is: Would another type of stimulation (or conventional practice) produce the same type of change as music over the same amount of time? Consequently, prudence is required in interpreting the results of studies using those designs. Third, single-subject designs alternating baseline conditions with intervention phases in which music stimulation is available (or extensions of those designs) are considered adequate to determine whether the music is responsible for changes in patient’s responding (i.e., of whether the patient is learning the association between the response and the music and using the response purposefully). Notwithstanding the adequacy of the designs, the number of patients involved in the studies using those designs was rather small and additional evidence is required to prove the dependability of the findings [73].

### 4.4. Future Research Directions

Future research may need to address and clarify a number of issues. First, replication efforts should be made to determine whether the results of some of the more prominent studies assessing the impact of recorded music or interactive music could be reproduced using methodologically sound research designs [74]. Second, a series of new studies may need to be carried out in which measures of both physiological indices and brain activity are collected to determine what types of correlations exist between those measures and thus understand how confidently those measures could be used and interpreted. Third, studies may be necessary to verify (a) whether interactive music is more effective than recorded music and, in case it is more effective, (b) what are the variables that make it so. Fourth, studies may also be required to determine the relationship that exists between (a) the increase of a specific response under a response-contingent music condition (i.e., increase indicating signs of awareness, non-reflective consciousness [35]), (b) the performance scores in a scale such as the Coma Recovery Scale-Revised, and (c) the behavioral responses appearing under conditions of recorded or interactive music. Only by pursuing these research objectives, one may be able to acquire functional knowledge essential to answer some of the questions that preclude the formulation of general statements about the impact of music and the definition of music-based intervention programs.

### 4.5. Limitations

Three limitations of this paper may be underlined here. First, a literature search restricted to articles written in English may prevent the detection and inclusion of relevant studies, which were published in other languages. Second, a search focused on three databases (i.e., PubMed, Web of Science, and PsycInfo) might be viewed as not sufficiently comprehensive and thus at risk of failing to identify some of the articles available in the area. With regard to this point, one could argue that (a) the combination of three main academic databases, as used for this review, is quite likely to guarantee an acceptable outcome [75], and (b) the search of the references of the cited literature as well as the use of search alert strategies throughout the writing of this paper may represent reasonable remedies to mitigate possible shortcoming in accessing relevant material. A third possible limitation is that no attempt was made to examine and summarize the data in an aggregated form to provide a quantitative synthesis of the studies through a meta-analysis [76]. The decision to present the data relying only on the use of a narrative approach was made in light of the many differences characterizing the studies between and within the three groups identified, and thus the view that a meta-analysis could provide an inaccurate/biased picture of the evidence available [77,78].

## 5. Conclusions

The present review was intended to provide a broad and informative picture of the research work conducted during the last decade to assess the impact of music on behavioral or non-behavioral responses of people with disorders of consciousness. Thirty-four studies were included in the review. They were grouped into three categories: (i) studies assessing the effects of recorded music, (ii) studies assessing the effects of interactive music, and (iii) studies assessing the effects of response-contingent music.

While the studies of all three categories reported fairly positive/encouraging results, several methodological questions make it difficult to draw conclusions about those results and their implications for intervention programs in daily contexts. For example, the studies assessing recorded music varied largely in the music and non-music stimulation conditions used, (b) the presentation time of those conditions, and (c) the types of measures adopted to determine the effects of music stimulation. The studies of the other two categories relied on multiple music sessions and sequential checks (i.e., without control conditions) or involved a relatively small number of patients.

Future research would need to pursue a number of critical objectives. For example, replication studies with methodologically sound designs should be carried out to determine the generality of some of the data available within each of the approaches. Studies comparing the impact of recorded music with the impact of interactive music would be essential to establish the differences between those approaches and the reasons/variables that are responsible for any such differences. Studies should also be designed to investigate the relationship between some of the measures used to determine the impact of music (e.g., physiological indices and measures of brain activity). Only by pursuing those objectives, one may acquire essential knowledge on the relevance of music for people with disorders of consciousness and on ways to use music within daily rehabilitation programs.

## Data Availability

Not pertinent.
